# Design of a cyclic peptide targeting intracellular *Staphylococcus aureus*

**DOI:** 10.1186/s43556-026-00519-z

**Published:** 2026-07-29

**Authors:** Álvaro Mourenza, Jesús Llano-Verdeja, Pablo Castañera, Rakesh Krishnan, Alicia Vogelaar, Blanca Lorente-Torres, Sergio Fernández-Martínez, Helena Á. Ferrero, Jennica Zaro, Jesús F. Aparicio, Luis M. Mateos, Cesar de la Fuente-Nunez, Michal Letek

**Affiliations:** 1 Grupo EXPRELA, Instituto de Investigación Biomédica de A Coruña (INIBIC), As Xubias, A Coruña, Spain; 2 Centro Interdisciplinar de Química e Bioloxía (CICA), Universidade da Coruña, A Coruña, Spain; 3 Departamento de Biología Molecular, Área de Microbiología, Universidad de León, 24071 León, Spain; 4 Machine Biology Group, Departments of Psychiatry and Microbiology, Institute for Biomedical Informatics, Institute for Translational Medicine and Therapeutics, Perelman School of Medicine, University of Pennsylvania, Philadelphia, PA USA; 5 Departments of Bioengineering and Chemical and Biomolecular Engineering, School of Engineering and Applied Science, University of Pennsylvania, Philadelphia, PA USA; 6 Department of Chemistry, School of Arts and Sciences, University of Pennsylvania, Philadelphia, PA USA; 7 Penn Institute for Computational Science, University of Pennsylvania, Philadelphia, PA USA; 8 Department of Pharmacology and Pharmaceutical Sciences, USC Alfred E. Mann School of Pharmacy and Pharmaceutical Sciences, University of Southern California, Los Angeles, CA 90089 USA; 9 Genómica y Proteómica (INBIOMIC), Instituto de Biología Molecular, Universidad de León, 24071 León, Spain; 10 Instituto de Desarrollo Ganadero y Sanidad Animal (INDEGSAL), Instituto de Investigación Biosanitaria de León (IBIOLEÓN), Campus Universitario Vegazana, 24071 León, Spain

**Keywords:** MRSA, Cyclotides, Antimicrobial peptides, Docking and molecular dynamics, Intracellular infections

## Abstract

Methicillin-resistant *Staphylococcus aureus* (MRSA) remains a major clinical challenge, particularly intracellular MRSA infections are difficult to treat because antimicrobial agents must combine stability, host-cell access and bacterial target engagement. Cyclotides offer highly stable cyclic scaffolds for peptide engineering, but their use as intracellular antimicrobial protein inhibitors remains largely unexplored. Here, we engineered a cyclotide-grafted derivative of the antimicrobial peptide KTR by inserting it into the MCoTI-I scaffold, generating the cyclic construct MCo-KTR2. Molecular docking and molecular dynamics suggested potential interactions between MCo-KTR2 and the resistance-associated penicillin-binding protein PBP2a. Site-directed mutagenesis and fluorescence polarization assays indicated that specific residues contribute to binding in vitro. Although MCo-KTR2 displayed lower activity than linear KTR in standard MIC assays, cyclotide grafting increased serum stability by more than 30-fold and enhanced cellular uptake, colocalising with cytosolic *S. aureus* during infection. These properties were associated with improved activity against intracellular bacteria without detectable cytotoxicity or haemolytic activity. Furthermore, MCo-KTR2 showed higher antibacterial activity when combined with the membrane-active compound Visomitin as well as in combination with vancomycin and gentamicin. Together, these findings identify cyclotide grafting as a strategy to improve peptide stability and intracellular delivery, and support MCo-KTR2 as a scaffold for further optimization against intracellular MRSA infections.

**Supplementary Information:**

The online version contains supplementary material available at 10.1186/s43556-026-00519-z.

## Introduction

*Staphylococcus aureus* is a major cause of antibiotic-resistant infections worldwide. Although it asymptomatically colonises the skin and mucosal surfaces of 20–30% of healthy individuals, it can also cause diseases ranging from superficial skin infections to severe, life-threatening conditions [1]. Globally, *S. aureus* is responsible for nearly one million deaths annually and represents the leading bacterial cause of mortality in 135 countries [1]. While methicillin-susceptible *S. aureus* (MSSA) accounts for most of these deaths, methicillin-resistant *S. aureus* (MRSA) still causes more than 100,000 deaths each year and remains a major contributor to antimicrobial resistance-associated mortality worldwide [2, 3]. Current treatment relies on β-lactams for MSSA infections and agents such as vancomycin, daptomycin, or linezolid for MRSA, depending on the infection site and clinical setting. However, treatment efficacy is often compromised by the ability of *S. aureus* to survive in both extracellular and intracellular niches, where eukaryotic cell membranes can limit antibiotic access and promote bacterial persistence [4–6].

Several strategies have been explored to identify new anti-*S. aureus* agents, including drug repurposing [4, 7], natural products with antimicrobial activity [8], host-directed therapies that stimulate antimicrobial responses in infected cells [9, 10], and computational methods based on virtual screening and artificial intelligence (AI) [11, 12]. Among these, antimicrobial peptides (AMPs) represent a promising alternative owing to their broad-spectrum activity and diverse mechanisms of action, which frequently involve disruption of bacterial membranes [13, 14]. However, many naturally occurring AMPs from animals, insects, and plants are linear, arginine-rich molecules with poor proteolytic stability [15]. Cyclotides, by contrast, have emerged as attractive scaffolds to overcome these limitations. These small cysteine-rich backbone-cyclised peptides [15] contain a cyclic cystine knot motif that confers exceptional stability and protease resistance [16]. Moreover, their inter-cysteine loops can be engineered to incorporate bioactive sequences, thereby generating peptides with novel biological functions while retaining the favourable properties of the cyclotide scaffold [17–19]. Cyclotides have also been reported to exhibit low immunogenicity in biological systems [20]. Despite these advantages, their development is often constrained by the cost and synthesis complexity of conventional solid-phase peptide synthesis (SPPS) followed by native chemical ligation [21]. Recombinant expression platforms based on mini-inteins, such as GyrA, provide an attractive alternative by enabling the efficient production and cyclisation of engineered cyclotides in bacterial systems [22, 23].

Despite their exceptional stability and structural versatility, the development of cyclotide-based antimicrobials remains challenging. Previous studies have shown that grafting pore-forming antimicrobial peptides into cyclotides often reduces antimicrobial activity, likely owing to steric and conformational constraints that limit membrane interactions [18, 19]. However, the ability of cyclotides to constrain and present bioactive sequences in defined conformations offers an alternative strategy that enhances target-specific protein interactions rather than membrane disruption. This approach is particularly attractive for targeting penicillin-binding protein 2a (PBP2a), the principal determinant of methicillin resistance in *S. aureus* [24, 25]. Although PBP2a-targeting antibiotics such as ceftaroline and ceftobiprole have improved the treatment of MRSA infections, effective eradication of intracellular *S. aureus* remains difficult because many antimicrobial agents exhibit poor cellular penetration and limited activity against intracellular bacterial reservoirs [24, 26, 27].

Here, we developed a cyclotide-based antimicrobial peptide aimed at targeting intracellular *S. aureus*. To facilitate cyclotide engineering, we established a recombinant production platform based on the mini-intein GyrA, enabling the efficient generation and evaluation of novel cyclotide variants. Using this approach, we engineered MCo-KTR2, a cyclotide-grafted derivative of the previously described antimicrobial peptide KTR [28, 29], and characterised its biochemical properties, mechanism of action, and intracellular antimicrobial activity. Furthermore, we identified a combination with Visomitin that enhanced the activity of MCo-KTR2 during host cell infection, highlighting the potential of engineered cyclotides as stable antimicrobial scaffolds for combination therapies targeting both cell wall synthesis and membrane integrity in intracellular MRSA infections.

## Results

### MCo-KTR2 exhibits favourable structural and biochemical properties

**Fig. 1:**
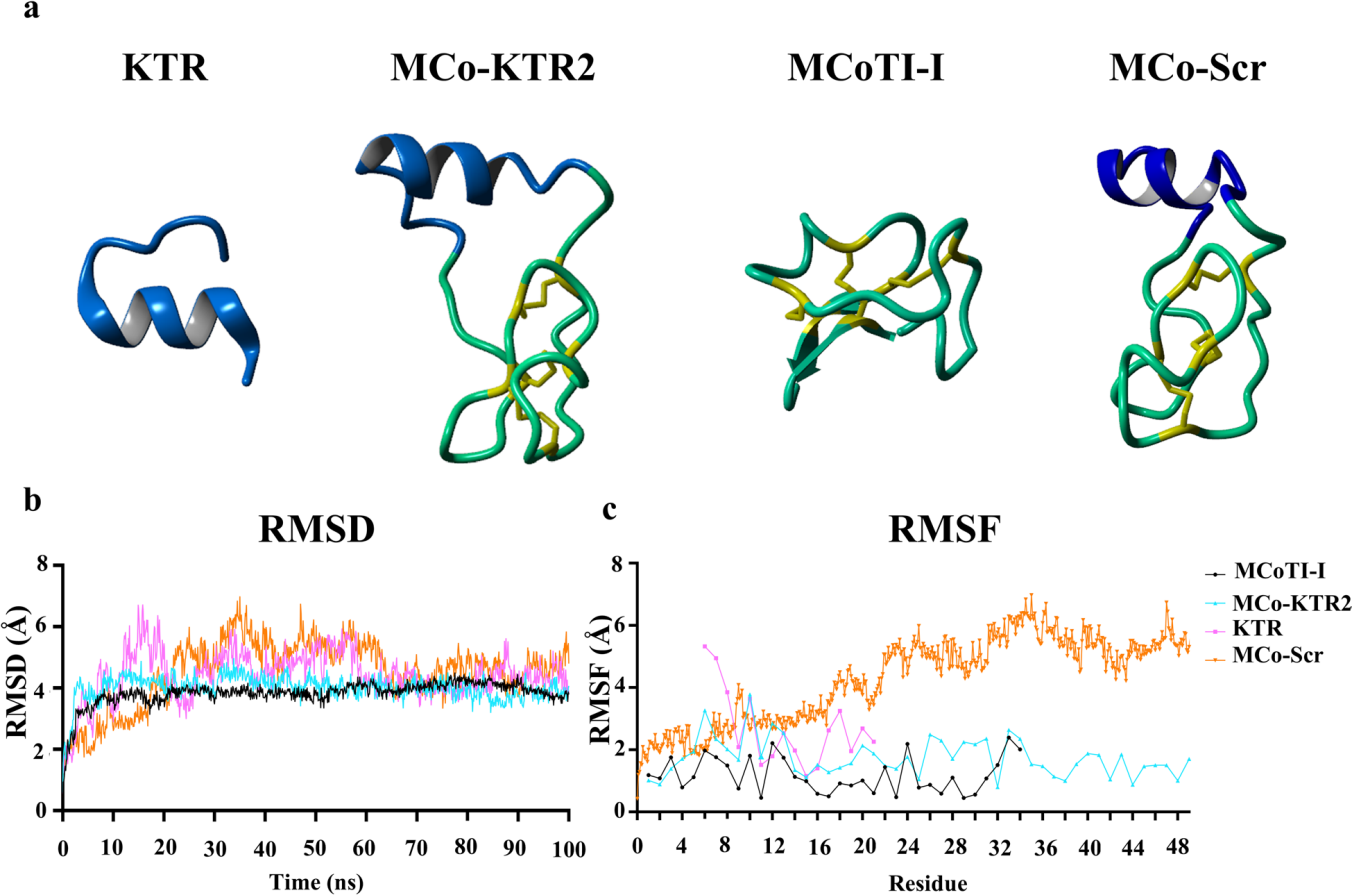
Peptide structures. **a** Theoretical in silico structures of KTR and MCo-KTR2 compared with the NMR-solved structure of MCoTI-I. KTR (blue) showed similar folding once grafted into MCo-KTR2 (green). Disulphide bonds and cysteine residues are highlighted in yellow. **b** RMSD of the peptides during a 100 ns molecular dynamics simulation. **c** RMSF of the four peptides following a 100 ns simulation

To generate a cyclotide-based antimicrobial peptide, we engrafted the previously described KTR sequence [28, 29], yielding the chimeric construct MCo-KTR2 (Table S1). Structural models were generated using SWISS-MODEL [30] and refined by energy minimization in YASARA Structure (version 20.7.4) [31, 32]. MCo-KTR2 was compared with linear KTR, a scrambled control peptide (MCo-Scr) grafted into the same scaffold and the MCoTI-I scaffold (Fig. 1a; Table S1).

Molecular dynamics simulations showed that the KTR sequence remains surface-exposed upon engraftment. Notably, the scrambled variant, MCo-Scr, also adopted a surface-exposed α-helical conformation, structurally similar to MCo-KTR2 (Fig. 1a). Despite this similarity, MCo-KTR2 exhibited greater structural stability, comparable to the native MCoTI-I scaffold, as indicated by lower root mean square deviation (RMSD) values over a 100 ns simulation (Fig. 1b). In particular, the N-terminal region of KTR was markedly stabilized upon grafting. In contrast, MCo-Scr displayed increased flexibility, reflected by higher root mean square fluctuation (RMSF) values and slightly reduced overall stability (Fig. 1c).

MCo-KTR2 was successfully expressed in soluble form in *E. coli* BL21(DE3) (Fig. S1). The linear precursor was cloned in frame with the Mxe GyrA intein and a chitin-binding domain to enable affinity purification, followed by on-column backbone cyclization and oxidative folding using MESNA [33, 34]. Expression was confirmed by SDS–PAGE (Fig. S1), and purity was assessed by RP–HPLC on a C18 column without additional purification steps. Chemically synthesized MCo-KTR2 (SPPS-derived) displayed an identical RP–HPLC elution profile to the recombinant peptide, confirming correct folding and cyclization (Fig. S2a,b).

**Fig. 2:**
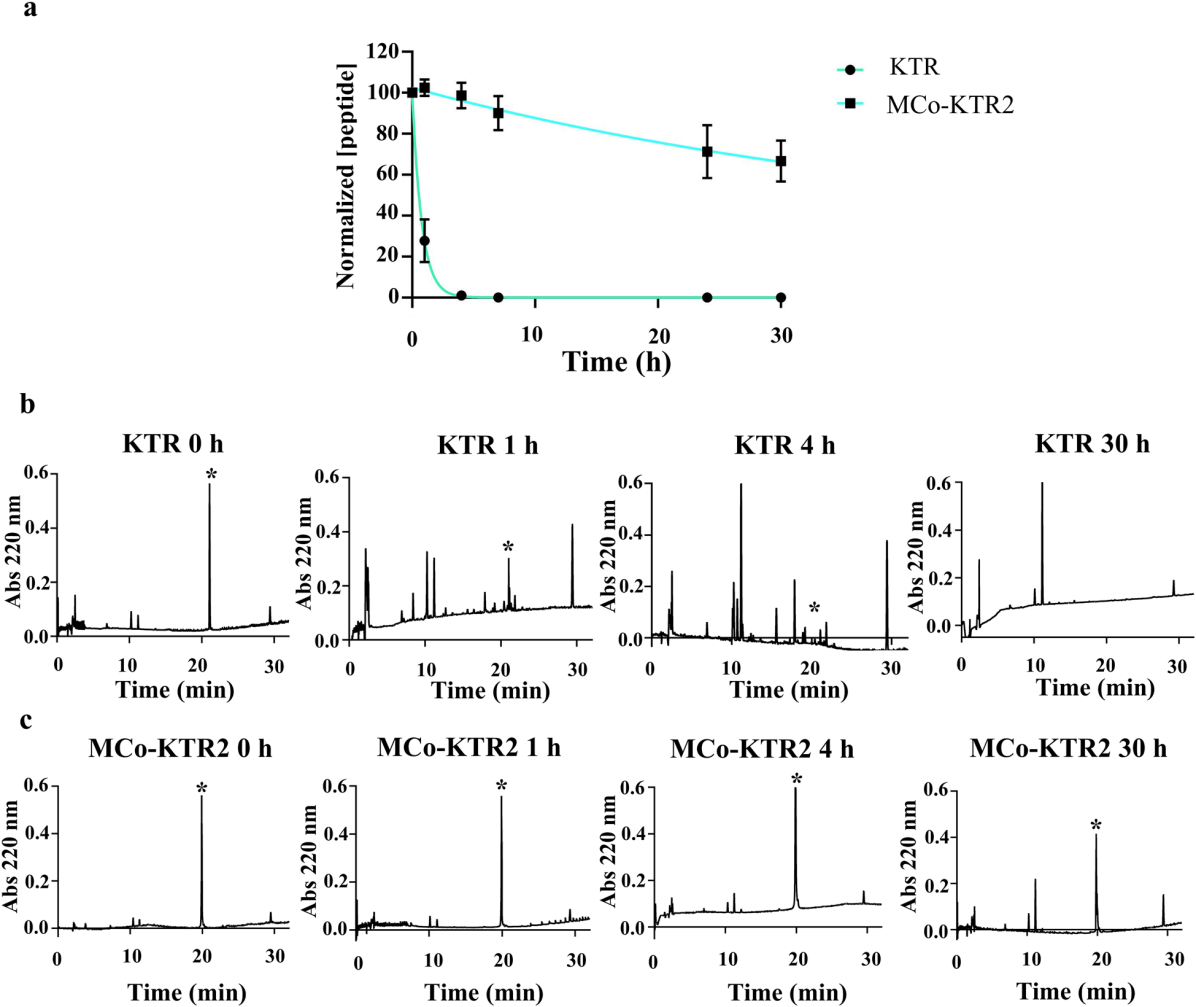
Serum stability of peptides. **a** Comparison of the serum stability of the cyclotide MCo-KTR2 and its linear precursor KTR over 30 h at 37 °C in human serum. The results represent the mean ± SD of three independent experiments. **b** Representative HPLC traces of KTR at different time points. **c** Representative HPLC traces of MCo-KTR2 at different time points. * Indicates the expected peptide peak

To evaluate the impact of engraftment on peptide stability, KTR and MCo-KTR2 were incubated in human serum at 37 °C and analysed by RP–HPLC over time (Fig. 2). KTR was rapidly degraded, with a half-life < 1 h and complete loss of the main peak after 4 h, accompanied by multiple degradation products (Fig. 2a,b). In contrast, MCo-KTR2 showed markedly enhanced stability, with a half-life > 30 h and only ~30% reduction of the main peak after 30 h, together with minor byproduct formation (Fig. 2a,c). These results show that cyclotide engraftment increases resistance to serum proteolysis by over 30-fold.

Collectively, these results demonstrate that engraftment of KTR into the MCoTI-I scaffold preserves a stable cyclotide fold, enables efficient recombinant production, and markedly improves resistance to serum proteolysis. These properties establish MCo-KTR2 as a structurally robust antimicrobial scaffold suitable for further functional evaluation.

### MCo-KTR2 displays selective antimicrobial activity against MRSA

To determine whether engraftment affected antimicrobial activity, we compared recombinant MCo-KTR2, synthetic MCo-KTR2, and linear KTR using broth microdilution assays in Cation-Adjusted Mueller–Hinton Broth (CAMHB) following CLSI guidelines [35]. Linear KTR showed the highest activity against *S. aureus* USA300 LAC (Community Acquired-MRSA, CA-MRSA; MIC = 6.3 µM), consistent with previous reports [29]. In contrast, MCo-KTR2 displayed reduced activity, with similar MIC values for both the recombinant (18.8 ± 8.8 µM) and synthetic peptide forms (16.7 ± 7.2 µM), approximately threefold higher than KTR (Table 1). The MCoTI-I scaffold alone showed no antimicrobial activity, as expected [19].

**Table 1:** Activity of the different peptides and antimicrobials against *S. aureus* USA300 LAC in CAMHB. The peptides were tested by the broth microdilution method, and the results represent the mean ± SD of three independent experiments with two technical replicates

Peptide/ Treatment	MIC (µM)	MIC + Visomitin 0.5 µM (µM)	MIC + Demeclocycline 0.1 µM (µM)	MIC + Vancomycin 0.9 µM (µM)	MIC + Tetracycline 1.6 µM (µM)	MIC + Gentamicin 3.1 µM (µM)
KTR	6.3 ± 0.0	3.3 ± 0.0	6.3 ± 0.0	0.6 ± 0.2	6.3 ± 0.0	1.1 ± 0.0
MCo-KTR2	18.8 ± 8.8	1.6 ± 0.0	18.8 ± 8.8	-	-	-
MCo-KTR2 synthetic	16.7 ± 7.2	1.6 ± 0.0	16.7 ± 7.2	0.3 ± 0.0	> 16.7	0.7 ± 0.3
MCoTI-I	> 100	> 100	> 100	-	-	-
Visomitin	2 ± 0	-	-	-	-	-
Demeclocycline	0.4 ± 0.0	-	-	-	-	-
Daptomycin	1.6 ± 0.0	-	-	-	-	-
Vancomycin	3.7 ± 0.0	-	-	-	-	-
Tetracycline	6.3 ± 0.0	-	-	-	-	-
Gentamicin	12.5 ± 0.0	-	-	-	-	-
Mutants	MIC	-	-	-	-	-
MCo-KTR3	> 50	-	-	-	-	-
MCo-KTR4	25 ± 0	-	-	-	-	-

We next evaluated the antimicrobial spectrum across additional strains. MCo-KTR2 retained activity against MRSA strains, with slightly improved potency against *S. aureus* NCTC 13626 (Hospital Acquired-MRSA, HA-MRSA; MIC = 12 µM), while showing limited or no activity against *S. aureus* ATCC 25923 (MSSA) and *E. coli* ATCC 25922 (Table S2). In contrast, KTR exhibited broader activity, including higher potency against MSSA (MIC = 1.8 ± 1.1 µM) and comparable activity against *E. coli* ATCC 25922 (MIC = 6.3 µM).

The reduced activity of MCo-KTR2 against non-PBP2a-expressing strains suggests increased target specificity following engraftment. These findings show that cyclotide grafting can generate an active antimicrobial peptide with selective activity against MRSA.

### MCo-KTR2 reduces peptidoglycan synthesis and binds PBP2a in vitro

**Fig. 3:**
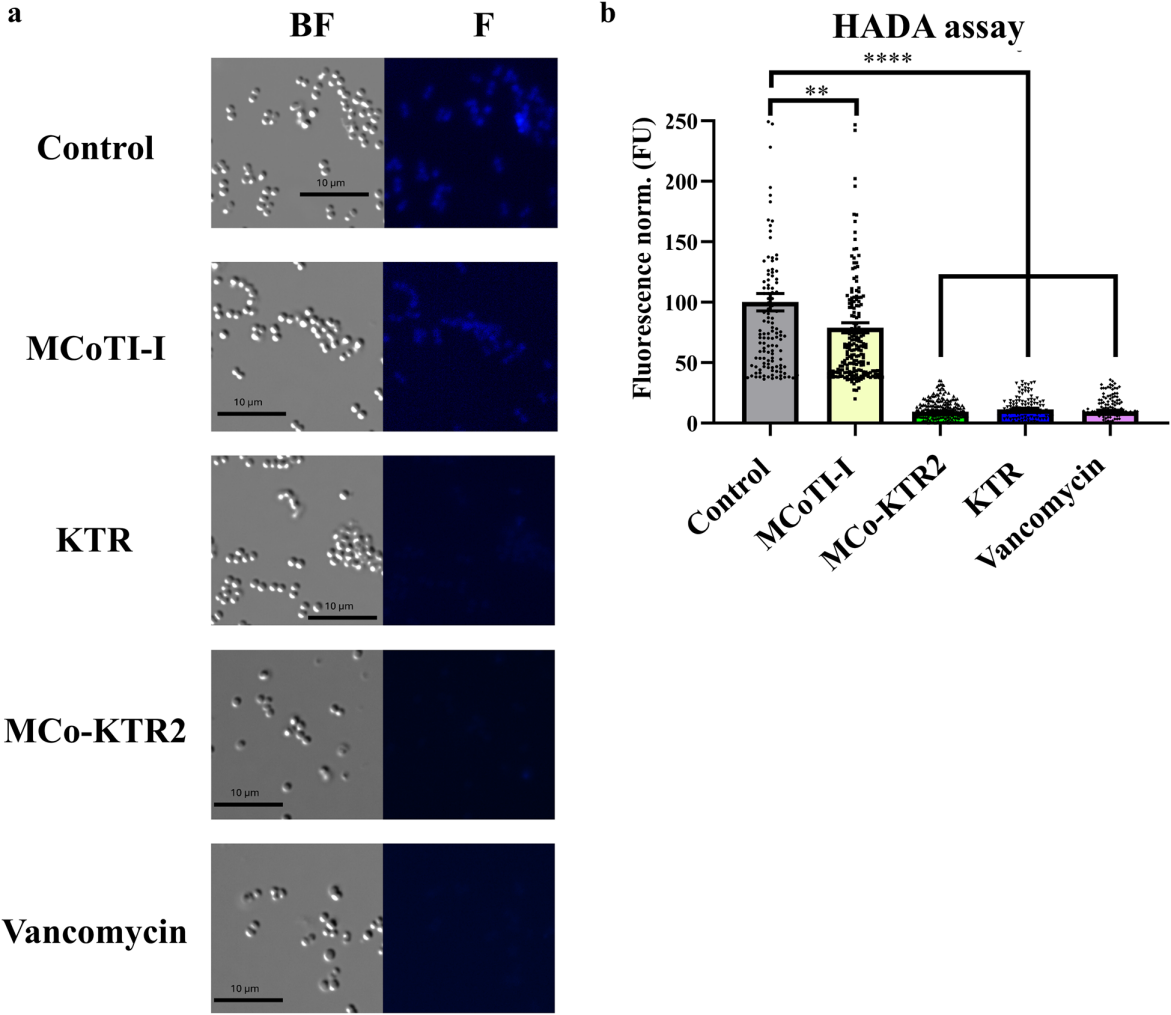
HADA assay of *S. aureus* USA300 LAC treated with 1/2 × MIC of the different peptides and vancomycin as a positive control. **a** Fluorescence microscopy images of *S. aureus* cells after treatment. **b** Quantitative image analysis of cellular fluorescence; data represent the fluorescence intensity of at least 150 cells from three independent experiments. As the data did not follow a normal distribution, the Mann–Whitney U test was used for group comparisons. ** p-value < 0.01; **** p-value < 0.0001

To determine whether engraftment affects KTR´s molecular mechanism of action, we first assessed cell wall synthesis using a 7-hydroxycoumarin-3-carboxylic acid–D-alanine (HADA) incorporation assay [36]. Treatment with vancomycin, MCo-KTR2 or linear KTR (1/2 × MIC) markedly reduced fluorescence compared with vehicle and MCoTI-I controls, indicating inhibition of peptidoglycan synthesis (Fig. 3a,b). No major morphological changes were observed by bright-field microscopy (Fig. 3a), consistent with previous reports for KTR [29]. These results confirm that MCo-KTR2 retains a cell wall–targeting mechanism.

**Fig. 4:**
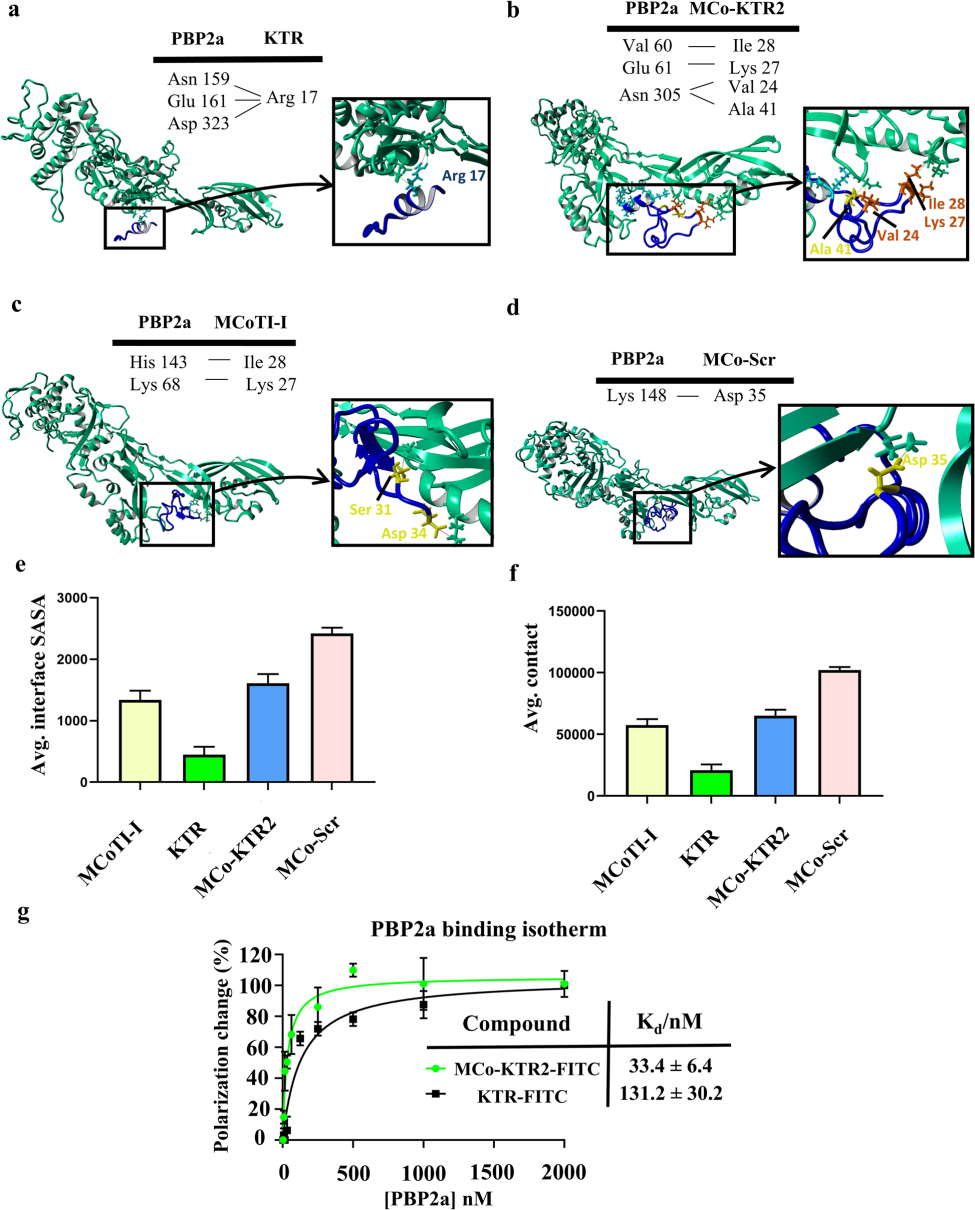
Peptides binding to PBP2a. **a** KTR (dark blue) docked to *S. aureus* PBP2a (green). Residues involved in side-chain contacts (light blue) are highlighted and listed in the table. **b** MCo-KTR2 (dark blue) docked to *S. aureus* PBP2a (green). Contact residues derived from the KTR segment (light blue) and from the cyclotide scaffold (yellow and orange) are shown and listed in the table; residues highlighted in orange were selected for mutagenesis. **c** MCoTI-I (dark blue) docked to *S. aureus* PBP2a (green). Scaffold-derived contact residues are highlighted in yellow and listed in the table. **d** MCo-Scr (scrambled peptide; dark blue) docked to *S. aureus* PBP2a (green). Residues originating from the MCoTI-I scaffold are highlighted in yellow; no stable peptide-derived contacts were observed after molecular dynamics simulations. **e** Average interface SASA of peptide–receptor complexes after equilibration. **f** Average number of receptor–peptide contacts. **g** The binding isotherm of PBP2a. FITC-labelled peptides (KTR-FITC and MCo-KTR2-FITC, 5 nM) were titrated with increasing concentrations of recombinant PBP2a. Fluorescence polarisation was measured using excitation at 480 nm and emission at 530 nm (parallel and perpendicular channels). Data represent mean ± SD of three independent experiments. Baseline polarisation was determined using free FITC titrated with PBP2a

Given that KTR has been described as a PBP2a binder [28, 29], we investigated the interaction of MCo-KTR2 with *S. aureus* PBP2a. Docking analyses were performed using the HADDOCK 2.4 server [37] with the PBP2a structure (PDB ID: 1MWT) [38], focusing on the allosteric domain (residues 137–310) [24, 25, 38]. Linear KTR was used as a positive control, whereas MCoTI-I and MCo-Scr served as negative controls. The most favourable docking solutions were further refined by 100 ns molecular dynamics (MD) simulations [39, 40] (Fig. 4a-d).

Initial docking suggested that all peptides could transiently occupy the allosteric pocket, likely due to their structural flexibility. However, MD simulations revealed that only KTR and MCo-KTR2 maintained stable interactions with PBP2a, whereas MCoTI-I and MCo-Scr rapidly lost contacts (Fig. S3; Fig. 4a–d). Interface analysis showed that MCo-KTR2 formed a larger and more stable interaction surface, with increased solvent-accessible surface area (SASA) and a higher number of receptor–peptide contacts compared with KTR (Fig. 4e,f). In contrast, MCoTI-I and MCo-Scr displayed broader but less specific interactions.

Consistent with these observations, binding affinity predictions using the PRODIGY server [41] indicated that MCo-KTR2 exhibited the strongest theoretical affinity, followed by KTR, MCo-Scr, and MCoTI-I (Table S3). These results suggest that while the cyclotide scaffold can accommodate the binding pocket, the KTR sequence is required for stable, specific target engagement, and that its engraftment enhances binding through additional scaffold-mediated contacts.

To experimentally validate these predictions, fluorescence polarisation assays were conducted using peptides fluorescently labelled with FITC (Fig. S4) and titrated against recombinant PBP2a. MCo-KTR2 displayed a significantly higher binding affinity than KTR, with a K_d_ of 33.4 ± 6.4 nM compared with 131.2 ± 30.2 nM for the linear peptide, indicating an approximately fourfold increase in binding upon cyclotide grafting and confirming that the MCoTI-I scaffold enhances target binding (Fig. 4g).

To further define the interaction interface, key residues identified by MD were mutated to alanine (V24A, K27A, I28A; MCo-KTR3), while additional mutations in non-interacting regions (D37A, P39A; MCo-KTR4) served as controls (Table S1). Recombinant peptides were expressed and purified under identical conditions, showing comparable profiles by HPLC and SDS–PAGE (Fig. S2). Functional analysis revealed that MCo-KTR3 exhibited markedly reduced antimicrobial activity, whereas MCo-KTR4 retained activity comparable to MCo-KTR2 (Table 1).

Overall, these findings indicate that MCo-KTR2 reduces peptidoglycan synthesis and support PBP2a binding as a plausible contributing mechanism, based on convergent functional, biophysical, and computational evidence. The identification of V24, K27, and I28 as critical binding determinants validates the predictive value of the in silico approach and demonstrates that cyclotide grafting enhances and stabilises target engagement.

### MCo-KTR2 activity is potentiated by the membrane-active compound Visomitin

To explore potential combinatorial effects, we evaluated MCo-KTR2 together with compounds showing different antibacterial mechanisms. In particular, we focused on Visomitin, a membrane-active anti-*S. aureus* compound [42] as well as antibiotics affecting cell wall (vancomycin) or protein synthesis (gentamicin, tetracycline, and demeclocycline).

While MCo-KTR2 (MIC = 18.8 ± 8.8 µM) and Visomitin (MIC = 2 µM) showed moderate activity as monotherapies, their combination resulted in a marked improvement, reducing the MIC of MCo-KTR2 to 1.6 ± 0.0 µM (Table 1). This effect was not observed with linear KTR, indicating that cyclotide engraftment is required for the enhanced response. No improvement was detected when either peptide was combined with tetracycline or demeclocycline, consistent with their shared mechanism of protein synthesis inhibition [43]. In contrast, combinations with vancomycin or gentamicin significantly enhanced activity, reducing MIC values to sub-micromolar levels (Table 1). Despite these strong effects, further analysis focused on Visomitin due to its relevance for intracellular infections and the lack of membrane-penetration activity of vancomycin and gentamycin [6, 44].

**Fig. 5:**
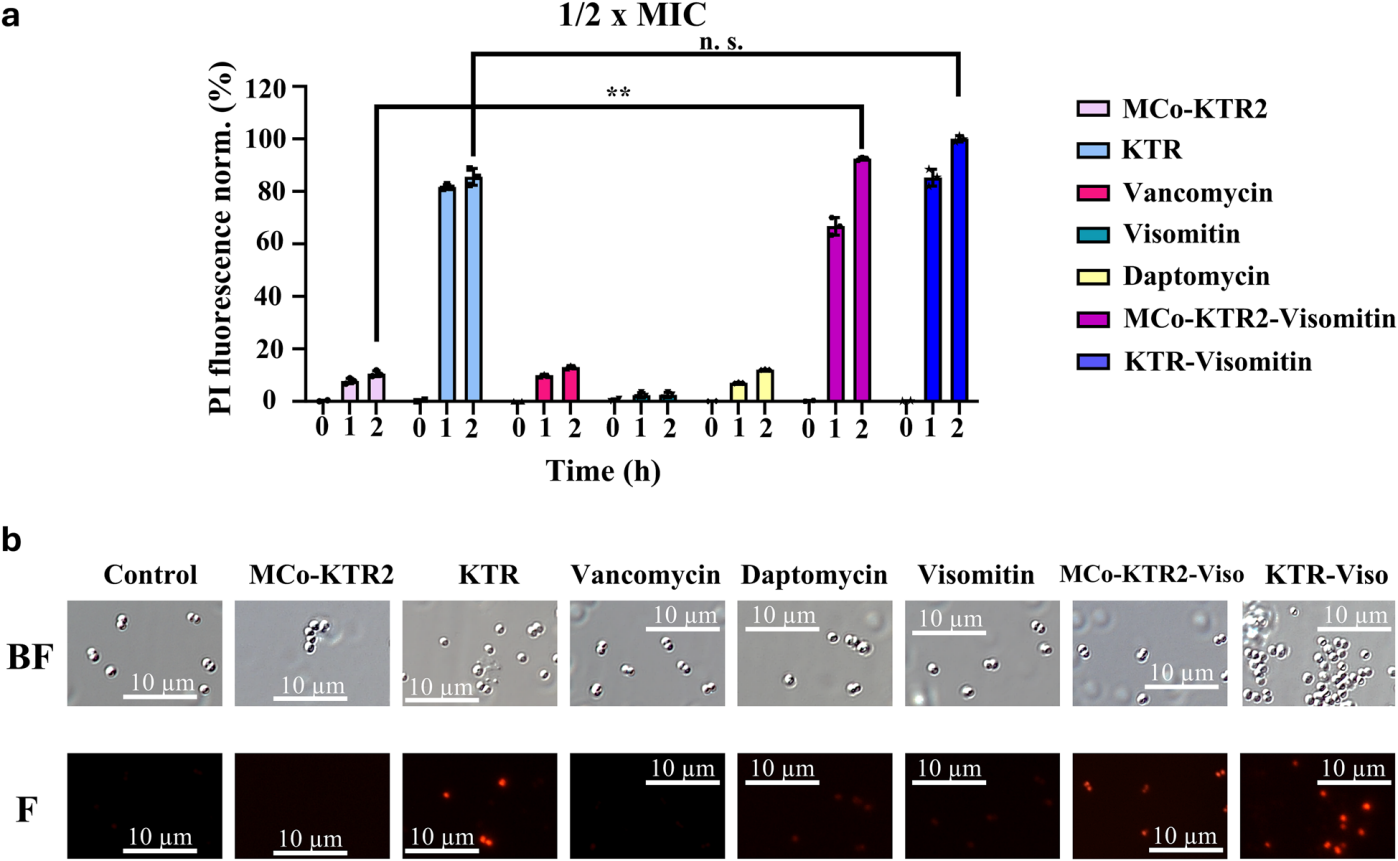
Propidium iodide uptake assay. **a** Plate reader fluorescence measurements of cells treated with different concentrations of the peptides and other antimicrobial compounds. Results are presented as mean ± SD from three independent experiments (*n* = 3), each performed with two technical replicates. Parametric analyses were performed, and Student’s *t*-test was used to compare treatments. ** *p*-value < 0.01. **b** Representative fluorescence microscopy images of *S. aureus* USA300 LAC following treatment with different antimicrobial compounds or combinations: MCo-KTR2 + Visomitin (MCo-KTR2–Viso) and KTR + Visomitin (KTR–Viso). The same field of view was imaged using bright-field (BF) and fluorescence (F) microscopy

To determine whether the enhanced antimicrobial effect of the MCo-KTR2–Visomitin combination involved membrane disruption as part of their mechanism of action, bacterial membrane integrity was analysed using a propidium iodide (PI) uptake assay [45, 46]. MCo-KTR2 alone did not measurably permeabilize bacterial membranes, whereas linear KTR significantly increased fluorescence, consistent with membrane disruption (Fig. 5a). Notably, KTR monotherapy produced a stronger permeabilizing effect than daptomycin, a clinically used membrane-active antibiotic [47]. Visomitin alone also showed no detectable effect under these conditions. However, the combination of MCo-KTR2 with Visomitin resulted in a pronounced increase in PI uptake within 1 h, indicating enhanced membrane permeabilization (Fig. 5). A similar trend was observed for KTR combined with Visomitin, although this effect was not significantly greater than KTR alone.

Fluorescence microscopy supported these findings. KTR-treated samples displayed heterogeneous fluorescence, whereas MCo-KTR2 combined with Visomitin produced a more uniform signal across the bacterial population (Fig. 5b), suggesting a broader effect despite lower average intensity. Therefore, MCo-KTR2 does not disrupt membranes on its own, but it seems to potentiate the membrane-disruptive activity of Visomitin. Together, these findings indicate that the combination enhances antibacterial activity, although the underlying mechanism remains to be established.

### MCo-KTR2 targets *S. aureus* during intracellular infections

*S. aureus* is a facultative intracellular pathogen [44, 48]. Notably, key antibiotics used to treat this organism, including vancomycin and gentamicin, display limited intracellular penetration [6, 44]. Therefore, the activity of the designed peptides was assessed in intracellular infection models.

To evaluate intracellular efficacy, we first assessed peptide cytotoxicity. Treatment of HEK293T cells with KTR, MCo-KTR2, or MCoTI-I (up to 100 µM) maintained > 90% cell viability, with minimal haemolytic activity observed at the highest concentrations (Fig. S5a,b), consistent with previous reports [28, 33].

**Fig. 6:**
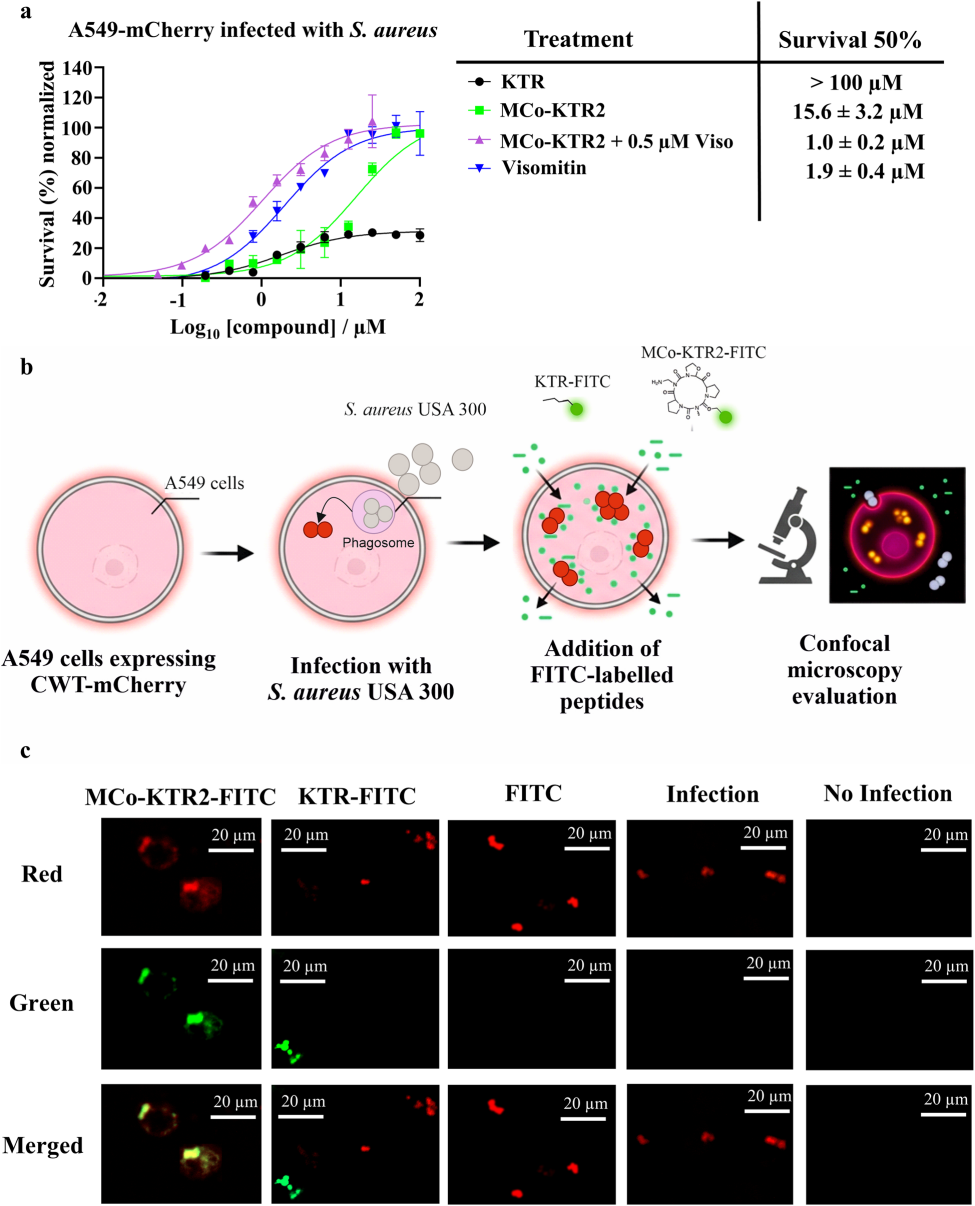
Intracellular activity and internalisation of peptides against *S. aureus* USA300 LAC in A549 cells. **a** Intracellular activity of peptides and combination therapies in mCherry-expressing A549 cells infected with *S. aureus* USA300 LAC (MOI = 10). Cells were infected for 1 h, treated with compounds at the indicated concentrations, and incubated for 24 h at 37 °C in 5% CO₂. mCherry fluorescence (580/625 nm) was used as a readout of infection. Data are shown as mean ± SD from three independent experiments and represent the concentration required to achieve 50% reduction in the infection-associated signal, normalised to vehicle control. **b** Schematic representation of the workflow used to assess peptide internalisation in infected cells. **c** Representative confocal microscopy images of CWT-mCherry A549 cells infected with *S. aureus* USA300 LAC and incubated with FITC-labelled peptides. Free FITC was used as a specificity control, and untreated infected cells (Infection) served as a bacterial labelling control. Non-infected cells were included to assess basal fluorescence

Intracellular antimicrobial activity was then evaluated using mCherry-expressing A549 cells [49] infected with *S. aureus* (MOI = 10). In the absence of treatment, infection resulted in complete host cell death at 24 h post-infection. Linear KTR showed negligible intracellular activity, failing to restore 50% cell viability even at 100 µM. In contrast, MCo-KTR2 significantly improved host cell survival, with an effective concentration of 15.6 ± 3.2 µM required to restore 50% viability (Fig. 6a), indicating retained antimicrobial activity against intracellular bacteria.

We next assessed combination effects with Visomitin. While Visomitin alone showed limited intracellular efficacy (EC₅₀ = 1.9 ± 0.4 µM), its combination with MCo-KTR2 markedly potentiated activity, lowering the effective concentration of MCo-KTR2 to 1.0 ± 0.2 µM in the presence of 0.5 µM Visomitin (Fig. 6a). Therefore, cyclotide engraftment confers intracellular activity, which is further enhanced by combination with a membrane-active compound.

To verify the intracellular uptake of the peptides, we performed fluorescence imaging using FITC-labelled peptides. Infected A549 cells expressing a cytosolic mCherry fused to a cell wall targeting domain of lysostaphin (CWT–mCherry) [50] were used to selectively label cytosolic *S. aureus* (Fig. 6b)*.* FITC-labelled MCo-KTR2 efficiently entered infected host cells and colocalised with intracellular *S. aureus* (Fig. 6c). In contrast, FITC-labelled KTR did not colocalise with intracellular bacteria and was primarily associated with extracellular bacteria. No signal was detected with free FITC, confirming specificity (Fig. 6c).

Together, these results demonstrate that MCo-KTR2 effectively penetrates eukaryotic cells, colocalises with intracellular *S. aureus*, and restores host cell viability. This intracellular activity, absent in the linear peptide, is further enhanced in combination with Visomitin.

## Discussion

The progressive decline in antibiotic discovery has intensified the search for alternative antimicrobial strategies. Antimicrobial peptides (AMPs) represent a promising class of therapeutics due to their structural diversity and multimodal mechanisms of action [51]. In addition to direct antibacterial effects, some AMPs exert immunomodulatory functions that can enhance host defence [52, 53]. However, their clinical translation remains limited by intrinsic drawbacks, including susceptibility to proteolysis, reduced stability in physiological environments, and the potential for resistance development through mechanisms such as protease secretion or membrane remodelling [54]. These limitations highlight the need for engineered peptide scaffolds with improved pharmacological properties.

In this study, we report the rational design of MCo-KTR2, a cyclotide-grafted derivative of the antimicrobial peptide KTR [28, 29], using the MCoTI-I scaffold [19, 55]. Cyclotides offer a structurally constrained and proteolytically stable framework that can be engineered to incorporate bioactive sequences. Consistent with this, molecular dynamics simulations indicated that grafting stabilises the KTR sequence, particularly at its termini, which are typically disordered in the linear peptide [15, 19, 56]. Experimentally, this translated into a > 30-fold increase in serum stability, confirming that cyclotide engraftment can effectively overcome one of the major limitations of linear AMPs [15, 18, 19, 33].

This gain in stability was accompanied by a moderate reduction in antimicrobial potency (~ threefold), a phenomenon frequently observed in grafted peptides [18, 19]. This likely reflects conformational constraints imposed by the cyclotide scaffold that limit membrane interactions [57]. However, rather than representing a simple loss of function, our data are consistent with a model in which reduced membrane disruption and PBP2a association may both contribute to activity. While linear KTR displays mainly membrane-disruptive activity, MCo-KTR2 exhibits reduced membrane-disruptive activity and enhanced inhibition of cell wall synthesis through PBP2a targeting. This is supported by HADA incorporation assays, docking, molecular dynamics analyses, mutagenesis and fluorescence polarisation measurements, which demonstrate that MCo-KTR2 binds PBP2a more strongly and stably than the linear peptide. Together, these findings support the hypothesis that binding to PBP2a contributes to the antimicrobial activity of MCo-KTR2, but further research will be required to determine the relative contribution of each mechanism of action.

Importantly, MCo-KTR2 displayed increased antimicrobial activity when used in combination with the membrane-active compound Visomitin. While MCo-KTR2 appears to inhibit peptidoglycan synthesis, Visomitin disrupts membrane integrity [42], and their combination resulted in enhanced membrane permeabilization and lower MIC values than either compound alone. Notably, this effect was more pronounced for MCo-KTR2 than for linear KTR, suggesting that the cell wall targeting activity of MCo-KTR2 may contribute to the enhanced antibacterial activity observed in combination with Visomitin [52, 58, 59].

Cyclotide engraftment appears to confer an intracellular advantage. This represents a major therapeutic challenge, as many antibiotics exhibit limited cellular penetration [6, 43]. MCo-KTR2 showed cellular penetration, rescued host cell viability, and colocalised with intracellular bacteria, whereas linear KTR showed no evidence of these effects. These differences may arise from the physicochemical properties of the cyclotide scaffold, which can facilitate cellular uptake while maintaining low cytotoxicity [56, 60]. Consistently, MCo-KTR2 showed minimal cytotoxicity at the highest concentration tested (> 100 µM) and intracellular activity at lower micromolar concentrations (~15 µM), suggesting a preliminary in vitro selectivity window of approximately tenfold [61]. However, we cannot exclude the possibility of additional indirect antimicrobial effects resulting from MCo-KTR2 treatment in human cells.

From a translational perspective, cyclotides provide a versatile platform for peptide engineering, combining high stability, structural adaptability, and the ability to incorporate functional sequences. The recombinant production strategy used here further enables scalable synthesis and rapid screening of variants, offering advantages over traditional chemical synthesis for iterative optimisation.

Nevertheless, several limitations should be acknowledged. Although we provide evidence for PBP2a binding, the mechanism remains partially indirect, and additional structural or biochemical studies would be required to fully resolve the interaction at atomic resolution. Furthermore, while the in vitro and cellular assays demonstrate the biological activity of MCo-KTR2, in vivo validation and additional preclinical studies are still required. Pharmacokinetic, biodistribution, and efficacy studies in relevant infection models will be necessary to determine its translational potential. Finally, although targeting the allosteric domain of PBP2a may impose a higher barrier to resistance development [62, 63], the long-term evolutionary response of bacteria to this strategy remains to be determined.

In conclusion, our results show that cyclotide grafting can improve the serum stability and cellular uptake of a linear antimicrobial peptide while preserving measurable anti-staphylococcal activity. The improved activity observed in combination with membrane-active compounds supports further exploration of cyclotide grafting as a peptide-engineering strategy for intracellular antibacterial applications.

## Materials and methods

### In silico design and analysis of peptides

The KTR peptide [29] was engrafted into loop 6 of the MCoTI-I scaffold to generate MCo-KTR2. Structural models were built using SWISS-MODEL and refined with the *md_refine.mcr* macro in YASARA [31, 32], including disulfide bond assignment and energy minimisation. Molecular dynamics (MD) simulations were performed for 100 ns at 310 K under physiological conditions, with trajectories recorded every 100 ps to assess structural stability [39, 40].

Docking analyses were conducted using HADDOCK 2.4 [37] with the *S. aureus* PBP2a structure (PDB: 1MWT). Based on previous studies [29], residues 137–310 of PBP2a were defined as the active region, while the full peptide sequences were included without restriction. The resulting complexes were subjected to all-atom MD simulations using NAMD3 with the CHARMM36m force field. Systems were solvated in a cubic water box (10 Å padding), neutralised with ions, minimised, and equilibrated to 310 K. Production runs were performed for 100 ns at 1 atm using a 2 fs timestep and a 12 Å cutoff for non-bonded interactions.

Binding affinities were estimated using PRODIGY [64] based on intermolecular contacts from MD-refined complexes.

### Bacterial strains

The following strains were used: *S. aureus* USA300 LAC (CA-MRSA), *S. aureus* NCTC 13626 (HA-MRSA), *S. aureus* ATCC 25923 (MSSA), *Escherichia coli* ATCC 25922, *E. coli* BL21(DE3), and *E. coli* DH5α. Bacteria were cultured on Tryptic Soy Agar (TSA) or Luria–Bertani (LB) agar, and in liquid media including Tryptic Soy Broth (TSB), cation-adjusted Mueller–Hinton broth (CAMHB), or 2 × YT, as appropriate for each assay.

### Chemical reagents

All reagents were purchased from Fisher Scientific unless otherwise indicated and were of HPLC or molecular biology grade. Chromatographic solvents were HPLC grade, and aqueous solutions were prepared using ultrapure water.

### Peptide cloning and expression

MCo-KTR2 and MCoTI-I were cloned into the pTXB1 vector (New England Biolabs), encoding an Mxe GyrA intein fused to a chitin-binding domain (CBD), as previously described [33]. The KTR peptide [29] was inserted into loop 6 of MCoTI-I, and constructs were codon-optimised for *E. coli*. DNA fragments containing NdeI and SapI sites (from Integrated DNA Technologies) were ligated into the digested vector and transformed into *E. coli* DH5α. Positive clones were identified by EcoRI screening and confirmed by Sanger sequencing.

For expression, plasmids were transformed into *E. coli* BL21(DE3). Cultures were grown in LB with ampicillin (100 µg/mL) at 37 °C to an OD₆₀₀ of ~0.6, induced with 1 mM IPTG, and incubated for 2 h. Cells were harvested by centrifugation and stored at −20 °C.

Cell pellets were resuspended in lysis buffer (50 mM Tris–Cl, 0.1 mM EDTA, 250 mM NaCl, 1% Triton X-100, 2% mannitol, 1 mM PMSF, pH 7.2) and lysed by sonication. After clarification (30,970 × *g*, 30 min), supernatants were incubated with chitin beads (2 mL per litre of culture) pre-equilibrated in column buffer. Beads were washed extensively, followed by a detergent-free buffer, and cyclotides were released by on-column intein cleavage and cyclisation using 250 mM MESNA (24 h, room temperature). Product quality was assessed by SDS-PAGE and RP-HPLC.

### Small-scale peptide expression testing

*E. coli* BL21(DE3) cells harbouring peptide constructs were grown overnight in LB at 37 °C with shaking (220 rpm), induced with 1 mM IPTG for 2 h, and harvested by centrifugation. Cells were lysed by freeze–thaw cycles, and clarified lysates were incubated with chitin beads (50 µL; New England Biolabs) for 20 min at 4 °C. After washing, bound fractions were analysed by SDS-PAGE to assess expression levels.

### Cyclotides desalting and lyophilisation

Cleaved cyclotides were desalted using C18 cartridges (Chromafix, Macherey–Nagel). After equilibration with acetonitrile and aqueous buffers, samples were acidified, loaded, washed, and eluted with 90% acetonitrile (ACN) containing 0.1% trifluoroacetic acid (TFA). Eluates were frozen, lyophilised, and resuspended in nuclease-free water.

### Minimum inhibitory concentration (MIC) assays

MICs were determined by broth microdilution in CAMHB as previously described [35]. Two-fold serial dilutions of compounds were prepared in 96-well plates and inoculated with exponentially growing *S. aureus* or *E. coli* (final concentration 2 × 10⁶ CFU/mL). Plates were incubated overnight at 37 °C under static conditions. MIC was defined as the lowest concentration preventing visible bacterial growth.

To assess combination effects, MICs of MCo-KTR2 were determined in the presence of subinhibitory concentrations (1/4 × MIC) of Visomitin (0.5 µM), demeclocycline (0.1 µM), vancomycin (0.9 µM), tetracycline (1.6 µM), or gentamicin (3.1 µM). Peptides were serially diluted in CAMHB and tested against *S. aureus* USA300 LAC under the same conditions. MICs of each compound alone were also determined.

Combination effects were expressed as the ratio:

$$CA=\left(\frac{MIC A }{MIC A+B}\right)$$

where CA (combined activity) reflects the change in peptide MIC in the presence of the second compound.

### Serum stability

Serum stability was assessed as previously described [19]. Briefly, 500 µg of linear precursor or cyclotide-derived peptide were incubated in 700 µL of clarified human serum at 37 °C. At defined time points (0, 1, 4, 7, 24, and 30 h), 100 µL aliquots were quenched with 900 µL of ACN containing 0.1% TFA, vortexed for 30 s, and incubated for 20 min at room temperature. Samples were centrifuged at 14,000 rpm for 15 min, and the supernatants were collected, lyophilised, and stored at −80 °C, along with the pellets, until analysis.

Prior to RP-HPLC, samples were reconstituted in H_2_O with 5% ACN and analysed on a C18 column. Peptide integrity was determined by peak integration, with time 0 defined as 100% peptide and all values normalised accordingly.

### HADA labelling of peptidoglycan

Peptidoglycan synthesis was visualised using HADA as previously described [36]. *S. aureus* precultures were diluted in 96-well plates containing TSB supplemented with peptides at 1/2 × MIC and incubated at 37 °C, 250 rpm for 2 h. Cultures were adjusted to OD₆₀₀ = 0.5 in TSB containing 250 µM HADA and incubated for 30 min at 37 °C with shaking. Cells were washed three times with PBS, mounted on glass slides, and analysed by fluorescence microscopy (excitation 350 nm, emission 460 nm).

### FITC labelling of peptides

Peptides were fluorescently labelled with fluorescein isothiocyanate (FITC isomer I, Merck) following established protocols with minor modifications [65]. Briefly, FITC (1.5 mg) was dissolved in 10 µL dimethylformamide (DMF). Peptides were dissolved at the highest achievable concentration in 250 µL of 1:1 0.1 M sodium carbonate buffer:DMF (pH 9). FITC was added at a tenfold molar excess, and reactions were incubated at room temperature in the dark under constant agitation. Conjugated peptides were purified by RP-HPLC in a C-18 column.

### Fluorescence polarisation binding assays

Fluorescence polarisation assays were performed as previously described [33] using FITC-labelled peptides to assess binding to recombinant PBP2a (ProteoGenix). To minimise potential effects of labelling on binding, equimolar mixtures of HPLC-purified fluorescent peaks (Fig. S4) were used at a final peptide concentration of 5 nM.

Measurements were performed at 22 °C using a Synergy XT plate reader (BioTek) with excitation at 480 nm and emission at 530 nm. Binding was assessed by titrating increasing concentrations of PBP2a in PBS supplemented with 1 mM EDTA and 4% (w/v) trehalose (pH 7.4). Free FITC controls showed no change in polarisation, and background fluorescence was subtracted using vehicle controls. Dissociation constants (K_d_) were calculated assuming a 1:1 binding model using GraphPad Prism.

### Site-directed mutagenesis

Site-directed mutagenesis of pTXB-MCo-KTR2 was performed using Platinum SuperFi DNA polymerase (Thermo Fisher Scientific) with mutagenic primers (Table S1). Reactions contained 100 ng template plasmid and 125 ng of each primer. Amplified products were digested with DpnI for 1 h at 37 °C to remove parental DNA and transformed into *E. coli* DH5α. Positive clones were verified by Sanger sequencing.

### Propidium Iodide (PI) uptake assay

Membrane integrity was assessed by propidium iodide uptake [45, 46]. Mid-log phase cultures (OD₆₀₀ = 0.5) were washed three times with PBS and exposed to compounds at 1/2, 1/4, and 1/8 × MIC for 0, 1, and 2 h. PI was then added to a final concentration of 10 µg/mL and incubated for 15 min. Fluorescence was measured using a VICTOR Nivo multimode plate reader (Revvity, Spain; excitation 530/30 nm, emission 625/30 nm). PBS-treated and PI-only samples served as controls.

For microscopy analysis, cells were concentrated to OD₆₀₀ = 1, treated with 1/2 × MIC for 1 h, and incubated with PI for 15 min in the dark. Samples were mounted on coverslips and visualised using fluorescence microscopy (excitation at 530 nm, emission at 620 nm).

### Haemolysis assay

Haemolytic activity was evaluated as previously described with minor modifications [19]. Defibrinated horse blood (Invitrogen) was washed three times in PBS (1,000 × g, 10 min). Peptide solutions (10 µL) were incubated with 90 µL of erythrocytes in PBS (final 4% v/v) at 37 °C for 1 h under agitation.

Samples were centrifuged (1,000 × g, 10 min), and haemoglobin release was quantified by measuring absorbance at 405 nm. PBS and 0.1% Triton X-100 were used as 0% and 100% haemolysis controls, respectively. Data were normalised to controls and represent three independent replicates per concentration.

### Infection assays of S. aureus in A549 cells

Human lung epithelial A549 cells (American Type Culture Collection, Ref. CCL-185) stably expressing mCherry were generated by transduction with pCDH-CMV-mCherry-T2A-Puro (Addgene #72,264) as previously described [49] and maintained in Dulbecco's Modified Eagle Medium (DMEM) supplemented with puromycin (Sigma-Aldrich, Spain) at 37 °C in 5% CO_2_. mCherry expression was routinely confirmed by fluorescence measurement (excitation 580 nm, emission 625 nm) using a VICTOR Nivo multimode plate reader (Revvity, Spain) [49]. Cell cultures were routinely tested for mycoplasma contamination.

For infection assays, 2 × 10^4^ cells per well were seeded in 96-well plates and infected with *S. aureus* at an MOI of 10 for 1 h at 37 °C. Subsequently, peptide monotherapies and combination treatments were added in a two-fold dilution series prepared in serum-free DMEM. Cells were incubated for 24 h, washed twice with PBS, and mCherry fluorescence was quantified (580/625 nm) as a readout of infection-associated signal.

### Intracellular targeting of bacteria with FITC-labelled peptides

Intracellular localisation of FITC-labelled peptides was assessed in *S. aureus*-infected A549 cells expressing CWT-mCherry as previously described [50]. Cells were seeded in glass-bottom 96-well plates and infected at an MOI of 10 as described above. After 1 h post-infection, a mixture of all HPLC-purified FITC-labelled peptide peaks at equal concentrations was added to the cultures. Final peptide concentrations were determined from absorbance at 280 nm and corrected using a factor of 0.3 [50]. Peptides were applied at 1/2 × MIC in serum-free medium without antibiotics, and cells were incubated for a further 3 h at 37 °C and 5% CO_2_.

Cells were imaged using a Zeiss LSM800 confocal microscope (Zeiss, Germany) equipped with Airyscan. CWT-mCherry-labelled intracellular bacteria were detected with excitation/emission at 587/610 nm, while FITC-labelled peptides were visualised at 490/530 nm. Both channels were acquired sequentially from the same field and merged for colocalisation analysis.

### Statistical analysis

Statistical analyses were performed using GraphPad Prism 8. Normality and homoscedasticity were assessed prior to conducting parametric tests. Differences between groups were evaluated using Student’s *t*-tests. Significance levels were defined as * *p*-value < 0.05; ** *p*-value < 0.01; *** *p*-value < 0.001; **** *p*-value < 0.0001. For datasets not meeting normality assumptions, non-parametric tests were applied as indicated in the respective figure legends.

## Supplementary Information

Supplementary Material 1.

Supplementary Material 2.

## Data Availability

Any additional information required to reanalyse the data reported in this paper and the plasmids generated in this work are available upon request.
