# Supplementary material for: Design of a cyclic peptide targeting intracellular Staphylococcus aureus
Source: Mol Biomed. 2026 Jul 29;7:120. doi: 10.1186/s43556-026-00519-z (PMC13421713; doi:10.1186/s43556-026-00519-z)
Supplement: Supplementary file 1 — Supplementary Material 1. [file 43556_2026_519_MOESM1_ESM.docx]

**Design of a cyclic peptide targeting intracellular *Staphylococcus aureus***

Álvaro Mourenza^*1,2+^, Jesús Llano-Verdeja^3^, Pablo Castañera^3^, Rakesh Krishnan^4,5,6,7^, Alicia Vogelaar^8^, Blanca Lorente-Torres^3^, Sergio Fernández-Martínez^3^, Helena Á. Ferrero^3^, Jennica Zaro^8^, Jesús F. Aparicio^3^, Luis M. Mateos^3,9^, Cesar de la Fuente-Nunez^4,5,6,7^, Michal Letek^*3,10^.

^1^ Grupo EXPRELA, Instituto de Investigación Biomédica de A Coruña (INIBIC), A Coruña, As Xubias, Spain

^2^Centro Interdisciplinar de Química e Bioloxía (CICA), Universidade da Coruña, Campus de Elviña, As Carballeiras, s/n, 15071 A Coruña, Spain. EXPRELA group.

^3^Departamento de Biología Molecular, Área de Microbiología, Universidad de León, 24071, León, Spain

^4^Machine Biology Group, Departments of Psychiatry and Microbiology, Institute for Biomedical Informatics, Institute for Translational Medicine and Therapeutics, Perelman School of Medicine, University of Pennsylvania, Philadelphia, Pennsylvania, United States of America.

^5^Departments of Bioengineering and Chemical and Biomolecular Engineering, School of Engineering and Applied Science, University of Pennsylvania, Philadelphia, Pennsylvania, United States of America.

^6^Department of Chemistry, School of Arts and Sciences, University of Pennsylvania, Philadelphia, Pennsylvania, United States of America.

^7^Penn Institute for Computational Science, University of Pennsylvania, Philadelphia, Pennsylvania, United States of America.

^8^Department of Pharmacology and Pharmaceutical Sciences, USC Alfred E. Mann School of Pharmacy and Pharmaceutical Sciences, University of Southern California, Los Angeles, CA 90089, USA.

^9^Instituto de Biología Molecular, Genómica y Proteómica (INBIOMIC), Universidad de León, 24071, León, Spain

^10^Instituto de Desarrollo Ganadero y Sanidad Animal (INDEGSAL), Instituto de Investigación Biosanitaria de León (IBIOLEÓN), Campus Universitario Vegazana, 24071, León, España.

^+^ Lead contact

***Corresponding Authors:** Álvaro Mourenza: [alvaro.mourenza@udc.es](mailto:alvaro.mourenza@udc.es); Michal Letek: [michal.letek@unileon.es](mailto:michal.letek@unileon.es).

**
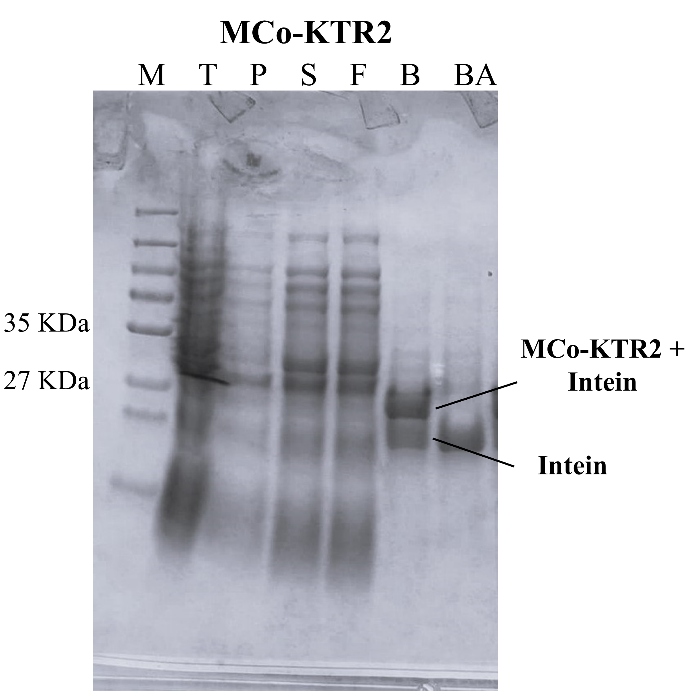
**

**Fig. S1** SDS–PAGE expression profile of MCo-KTR2 in *E. coli* BL21 (DE3).
M: molecular weight marker; T: total cell lysate; P: pellet fraction; S: soluble fraction; F: unbound fraction; B: protein bound to chitin beads; BA: protein content after cyclotide thiolation and elution.


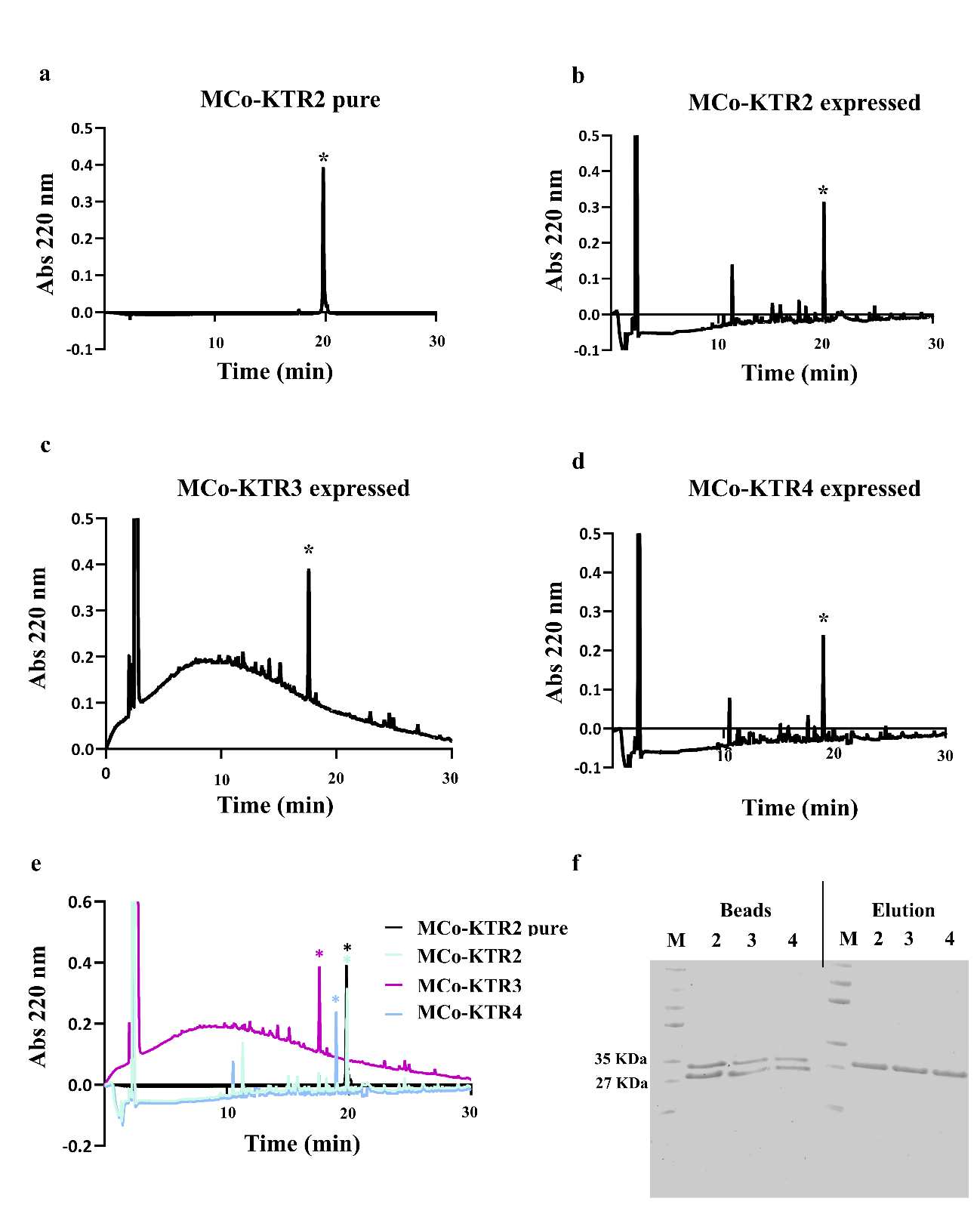


**Fig. S2** Expression and comparison of different mutants and peptides.
(a–d) RP–HPLC profiles of the different peptides: (a) MCo-KTR2 synthesised chemically; (b) MCo-KTR2 expressed in *E. coli*; (c) MCo-KTR3 expressed in *E. coli*; (d) MCo-KTR4 expressed in *E. coli*. (e) Superposition of the chromatographic profiles to compare purity. (f) SDS–PAGE comparison of peptide purity before bead purification and after thiolation/elution. M: molecular weight marker, 2: MCo-KTR2; 3: MCo-KTR3; 4: MCo-KTR4.

**
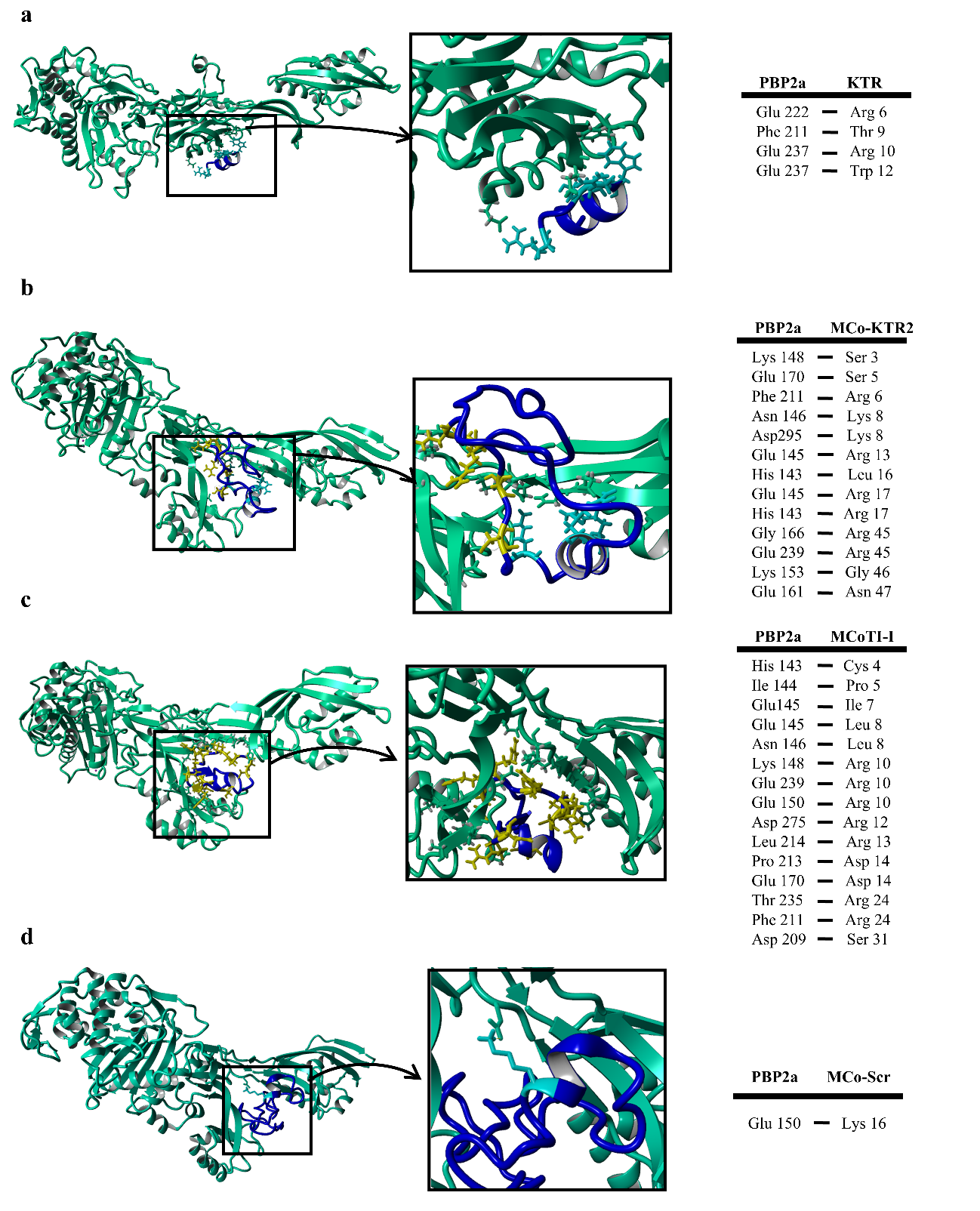
**

**Fig. S3** Docking structures obtained prior to MD simulations. (a) KTR (dark blue) docked to *S. aureus* PBP2a (green). Contact residues from KTR are highlighted in light blue. (b) MCo-KTR2 (dark blue) docked to *S. aureus* PBP2a (green). Contact residues derived from KTR are highlighted in light blue, and those from the MCoTI-I scaffold are shown in yellow. (c) MCoTI-I (dark blue) docked to *S. aureus* PBP2a (green). Contact residues from MCoTI-I are highlighted in light yellow. All contact residues for each peptide are included in the tables. (d) MCo-Scr (dark blue) docked to *S. aureus* PBP2a (green). Contact residues from MCo-Scr are highlighted in light blue.


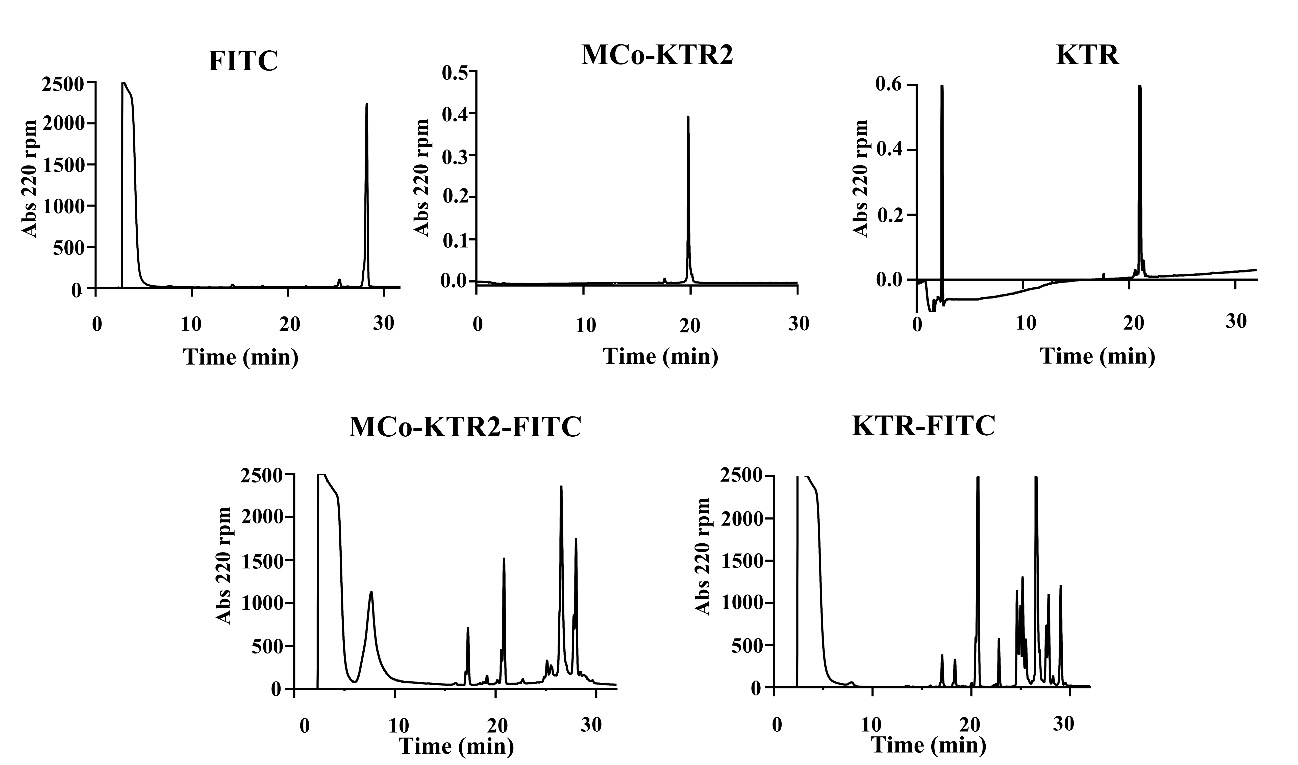


**Fig. S4** HPLC traces of FITC-labelled peptides. The upper row shows the HPLC traces of free FITC and the unlabelled peptides MCo-KTR2 and KTR. The lower row shows the HPLC peaks corresponding to the FITC-labelled peptide species.

**
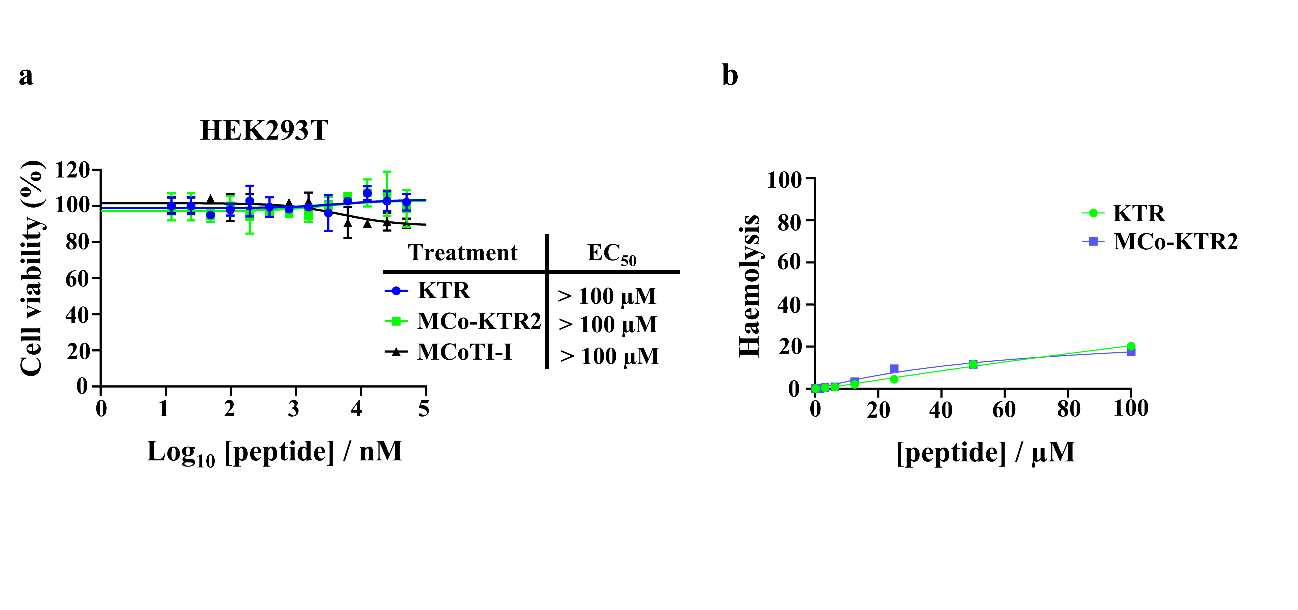
 Fig. S5** Peptides, cell toxicity, and haemolytic activity**.** (a) MTT assay assessing cytotoxicity of KTR, MCo-KTR2, and MCoTI-I in HEK293T human cells. (b) Haemolytic analysis of KTR and MCo-KTR2 against horse red blood cells. Results represent mean ± SD of three independent experiments.

**Table S1:** Primer and peptide sequences. Mutations in the peptides are highlighted in bold, and the KTR sequence is underlined.

| **Primer name** | **Sequence** | **Reference** |
| --- | --- | --- |
| MCoTI-I-Fw | tatgtgcggcagcggcagcgatggcggtgtgtgcccgaaaattctgcagcgttgccgtcgcgatagcgattgcccgggtgcgtgtatttgtcgtggcaatggctat | ^1^ |
| MCoTI-I-Rv | gcaatagccattgccacgacaaatacacgcacccgggcaatcgctatcgcgacggcaacgctgcagaattttcgggcacacaccgccatcgctgccgctgccgcaca | ^1^ |
| MCo-KTR2-Fw | tatgtgcggcagcggcagccgcattaaaacccgcacctggcgcctggcgttacgctggctgaaactgggcggtgtgtgcccgaaaattctgcagcgctgccgccgcgatagcgattgcccgggcgcgtgcatttgccgcggcaacggctat | This study |
| MCo-KTR2-Rv | gcaatagccgttgccgcggcaaatgcacgcgcccgggcaatcgctatcgcggcggcagcgctgcagaattttcgggcacacaccgcccagtttcagccagcgtaacgccaggcgccaggtgcgggttttaatgcggctgccgctgccgcaca | This study |
| mutKTR3-Fw | gcggcagcgctgcagagctgccgggcacgcaccgcccagtttcag | This study |
| mutKTR3-Rv | ctgaaactgggcggtgcgtgcccggcagctctgcagcgctgccgc | This study |
| mutKTR4-Fw | gcacgcgcccgcgcaagcgctatcgcgg | This study |
| mutKTR4-Rv | ccgcgatagcgcttgcgcgggcgcgtgc | This study |
| **Peptide name** | **Peptide sequence** | **Reference** |
| MCoTI-I | CGSGSDGGVCPKILQRCRRDSDCPGACICRGNGY | ^1^ |
| MCo-Scr | CGSGSLWRKTLRIALKRTWLRGGVCPKILQRCRRDSDCPGACICRGNGY | This study |
| KTR | RIKTRTWRLALRWLKL | ^2^ |
| MCo-KTR2 | CGSGSRIKTRTWRLALRWLKLGGVCPKILQRCRRDSDCPGACICRGNGY | This study |
| MCo-KTR3 | CGSGSRIKTRTWRLALRWLKLGG**A**CP**AA**LQRCRRDSDCPGACICRGNGY | This study |
| MCo-KTR4 | CGSGSRIKTRTWRLALRWLKLGGVCPKILQRCRRDS**A**C**A**GACICRGNGY | This study |

**Table S2.** Activity of the different peptides tested against different *S. aureus* and *E. coli* strains in CAMHB. The peptides were tested by the broth microdilution method, and the results represent the mean ± SD of three independent experiments (n = 3), with 2 technical replicates each.

| **Peptide/Treatment** | **KTR** | **MCo-KTR2** |
| --- | --- | --- |
| *S. aureus* NCTC 13626 (MRSA) | 6.3 ± 0.0 µM | 12.5 ± 0.0 µM |
| *S. aureus* ATCC 25923 (MSSA) | 1.8 ± 1.1 µM | 50 ± 0.0 µM |
| *E. coli* ATCC 25922 | 6.3 ± 0.0 µM | > 50 µM |

**Table S3:** Theoretical K_d_ of the different peptides, calculated using PRODIGY.

| **Peptide** | **Theoretical K_d_ for peptide-PBP2a binding** |
| --- | --- |
| KTR | 3 x 10^-7^ M |
| MCoTI-I | 1.9 x 10^-5^ M |
| MCo-KTR2 | 1.7 x 10^-10^ M |
| MCo-Scrambled | 1.6 x 10^-7^ M |
