# Supplementary material for: Design of a cyclic peptide targeting intracellular Staphylococcus aureus
Source: Mol Biomed. 2026 Jul 29;7:120. doi: 10.1186/s43556-026-00519-z (PMC13421713; doi:10.1186/s43556-026-00519-z)
Supplement: Supplementary file 2 — Supplementary Material 2. [file 43556_2026_519_MOESM2_ESM.docx]

**Design of a cyclic peptide targeting intracellular *Staphylococcus aureus***

Álvaro Mourenza^*1,2+^, Jesús Llano-Verdeja^3^, Pablo Castañera^3^, Rakesh Krishnan^4,5,6,7^, Alicia Vogelaar^8^, Blanca Lorente-Torres^3^, Sergio Fernández-Martínez^3^, Helena Á. Ferrero^3^, Jennica Zaro^8^, Jesús F. Aparicio^3^, Luis M. Mateos^3,9^, Cesar de la Fuente-Nunez^4,5,6,7^, Michal Letek^*3,10^.

^1^ Grupo EXPRELA, Instituto de Investigación Biomédica de A Coruña (INIBIC), A Coruña, As Xubias, Spain

^2^Centro Interdisciplinar de Química e Bioloxía (CICA), Universidade da Coruña, Campus de Elviña, As Carballeiras, s/n, 15071 A Coruña, Spain. EXPRELA group.

^3^Departamento de Biología Molecular, Área de Microbiología, Universidad de León, 24071, León, Spain

^4^Machine Biology Group, Departments of Psychiatry and Microbiology, Institute for Biomedical Informatics, Institute for Translational Medicine and Therapeutics, Perelman School of Medicine, University of Pennsylvania, Philadelphia, Pennsylvania, United States of America.

^5^Departments of Bioengineering and Chemical and Biomolecular Engineering, School of Engineering and Applied Science, University of Pennsylvania, Philadelphia, Pennsylvania, United States of America.

^6^Department of Chemistry, School of Arts and Sciences, University of Pennsylvania, Philadelphia, Pennsylvania, United States of America.

^7^Penn Institute for Computational Science, University of Pennsylvania, Philadelphia, Pennsylvania, United States of America.

^8^Department of Pharmacology and Pharmaceutical Sciences, USC Alfred E. Mann School of Pharmacy and Pharmaceutical Sciences, University of Southern California, Los Angeles, CA 90089, USA.

^9^Instituto de Biología Molecular, Genómica y Proteómica (INBIOMIC), Universidad de León, 24071, León, Spain

^10^Instituto de Desarrollo Ganadero y Sanidad Animal (INDEGSAL), Instituto de Investigación Biosanitaria de León (IBIOLEÓN), Campus Universitario Vegazana, 24071, León, España.

^+^ Lead contact

***Corresponding Authors:** Álvaro Mourenza: [alvaro.mourenza@udc.es](mailto:alvaro.mourenza@udc.es); Michal Letek: [michal.letek@unileon.es](mailto:michal.letek@unileon.es).


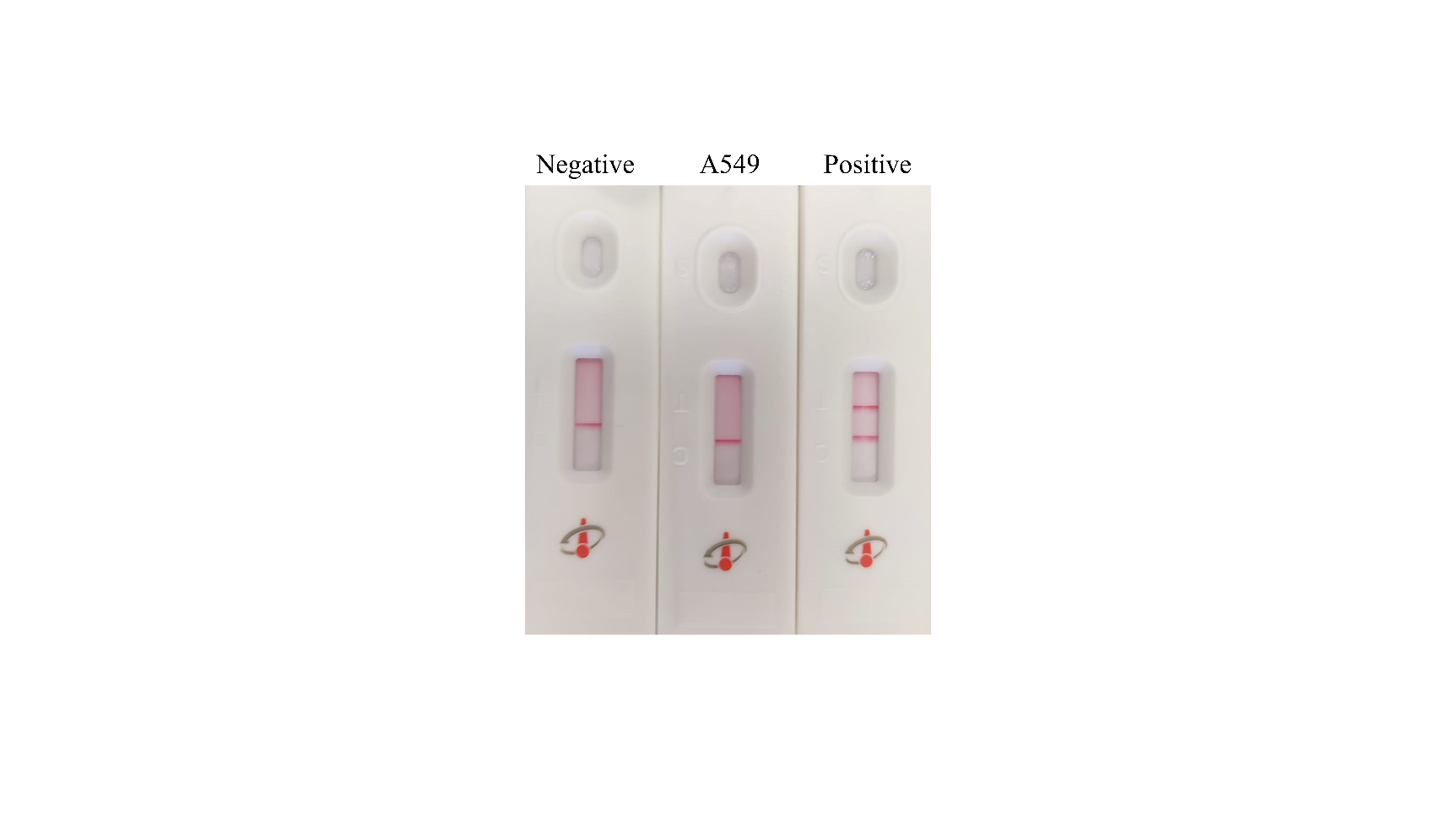


**Fig. S6.** Mycoplasma testing of A549 cells. Representative routine mycoplasma test showing the positive control, negative control, and A549 cell sample. The A549 cells tested negative for mycoplasma contamination.
